# Learning curve of robotic-assisted splenic vessel-preserving spleen-preserving distal pancreatectomy by one single surgeon: a retrospective cohort study

**DOI:** 10.1186/s12893-023-02294-y

**Published:** 2023-12-19

**Authors:** Xi-Tai Huang, Jin-Zhao Xie, Jian-Peng Cai, Wei Chen, Liu-Hua Chen, Xiao-Yu Yin

**Affiliations:** Department of Pancreato-Biliary Surgery, The First Affiliated Hospital, Sun Yat-sen University, 58 Zhongshan 2nd Rd, Guangzhou, Guangdong 510080 P. R. China

**Keywords:** Robotic-assisted surgery, Spleen-preserving distal pancreatectomy, Splenic vessels preservation, Learning curve

## Abstract

**Aim:**

Splenic vessel-preserving spleen-preserving distal pancreatectomy (SVP-SPDP) has a lower risk of splenic infarction than the splenicvessel-sacrificing SPDP, but it is more technically demanding. Learning curve of robotic-assisted SVP-SPDP (RSVP-SPDP) remains unreported. This study sought to analyze the perioperative outcomes and learning curve of RSVP-SPDP by one single surgeon.

**Methods:**

Seventy-four patients who were intended to receive RSVP-SPDP at the First Affiliated Hospital of Sun Yat-sen University between May 2015 and January 2023 were included. The learning curve were retrospectively analyzed by using cumulative sum (CUSUM) analyses.

**Results:**

Sixty-two patients underwent RSVP-SPDP (spleen preservation rate: 83.8%). According to CUSUM curve, the operation time (median, 318 vs. 220 min; *P* < 0.001) and intraoperative blood loss (median, 50 vs. 50 mL; *P* = 0.012) was improved significantly after 16 cases. Blood transfusion rate (12.5% vs. 3.4%; *P* = 0.202), postoperative major morbidity rate (6.3% vs. 3.4%; *P* = 0.524), and postoperative length-of-stay (median, 10 vs. 8 days; *P* = 0.120) improved after 16 cases but did not reach statistical difference. None of the patients had splenic infarction or abscess postoperatively.

**Conclusion:**

RSVP-SPDP was a safe and feasible approach for selected patients after learning curve. The improvement of operation time and intraoperative blood loss was achieved after 16 cases.

## Introduction

Spleen-preservation distal pancreatectomy (SPDP) is performed for benign or low-grade malignant tumors of the pancreatic body and tail [1, 2], which can be performed by using two surgical techniques. One is splenic vessel-preserving (SVP-SPDP) technique (the Kimura’s technique) where only the distal pancreas is resected while the splenic vein and artery are preserved [3]. Another one is splenic vessel-sacrificing (SVS-SPDP) technique (the Warshaw’s technique) that the preserved spleen is perfused with the left gastroepiploic and short gastric vessels, which is considered simpler [4]. SVP-SPDP was preferred because of the decreased risk of splenic infarction and gastric varices compared to SVS-SPDP [5, 6]. Nevertheless, SVP-SPDP was considered more difficult than SVS-SPDP technically, because rupture of a small splenic vessel branch may obscure the surgical field, resulting in massive intraoperative bleeding and inevitably innocent splenectomy.

Nowadays minimally invasive SPDP (MI-SPDP) includes robotic-assisted and laparoscopic SPDP. Robotic-assisted SPDP was reported to have a higher rate of spleen preservation than laparoscopic SPDP owing to its 3D high-definition views and dexterous manipulation with tremor-filtration [7, 8]. However, most of the studies about robotic-assisted SPDP included SVP-SPDP and SVS-SPDP [9–11], and the learning curve of robotic-assisted SVP-SPDP (RSVP-SPDP) remains undocumented. Therefore, the current study sought to analyze the postoperative outcomes and learning curve of RSVP-SPDP by one single surgeon in our institute.

## Materials and methods

### Patient selection and data definition

Patients who were planned to receive RSVP-SPDP from May 2015 to January 2023 at the First Affiliated Hospital of Sun Yat-sen University were included. This study was approved by the Ethics Committee of the First Affiliated Hospital of Sun Yat-sen University (Approval number: [2023]286).

Postoperative 30-day complication was evaluated according to the Clavien-Dindo classification [12]. Complications with severity ≥ Clavien-Dindo classification grade III were defined as major complications. The definition of postoperative pancreatic fistula (POPF) was determined according to the 2016 International Study Group of pancreatic surgery (ISGPS) definition [13]. Clinically relevant POPF (CR-POPF) included grade B and grade C POPF [14].

### Surgical technique

All procedures were performed by one single surgeon (Prof. Xiao-Yu Yin) by using da Vinci Si Surgical System (Intuitive Surgical, Inc, Sunnyvale, California, USA). The trocar placement was similar to the previously published article [15]. Robotic permanent cautery hook and Harmonic scalpel was used as the energy device.

The surgical procedure of RSVP-SPDP was as follow: The gastrocolic ligament was divided, the stomach was lifted by the 3rd robotic arm, and the pancreas was exposed. Intraoperative ultrasound was used to determine the location of the tumor and the resection margin if necessary. The inferior border of the pancreas was dissected, and the superior mesenteric vein (SMV) and splenic vein was then identified. The retropancreatic tunnel was established along SMV. The superior border of the pancreas, and the origin of splenic artery was identified. The posterior side of pancreatic neck was dissected, and the splenic vein was exposed. The pancreas was transected by ultrasonic scalpel or stapler, and the pancreatic stump was reinforced by interrupted U-suture. The pancreas was dissected and mobilized from right to left, and the collateral branches from the splenic vessels were clipped and divided. The pancreatic tail was carefully dissected from the splenic hilum. Then the pancreas was completely removed and put in a specimen bag. Drainage tube was placed close to the pancreatic stump for postoperative fluid amylases measurement. The specimen was extracted through the assistant port.

### Perioperative management and follow up

The prophylactic antibiotic was administered intraoperatively and can be administered up to 48 h postoperatively. Somatostatin or its analogs was administered postoperatively. Liquid diet was started on the postoperative day 1. The drains can be removed when the amylase level of drainage fluid was less than three times the normal upper limit. Otherwise, drains will be maintained until no drainage is present.

The patients were routinely followed up in the outpatient. Enhanced CT-scan was performed usually on the first postoperative follow up. If no significant abnormalities were found, scheduled ultrasound will be performed every 3–6 months and CT-scan will be performed every 6–12 months.

### Statistical analysis

All statistical analyses were performed with SPSS version 24.0 software (IBM, Inc, Armonk, NY). The categorical variables were presented as frequencies with percentages, while the continuous variables were presented as medians with interquartile range (IQR) or mean with range. Differences between categorical variables were compared by chi square test or Fisher’s exact test. Differences between continuous variables were compared by Mann-Whitney *U* test. The learning curve was described by using a cumulative sum (CUSUM) curve [16]. Two-tailed *P* < 0.05 was considered statistically significant.

## Results

### Clinicopathological features of patients undergoing robotic-assisted SVP-SPDP

Seventy-four patients who were planned to receive RSVP-SPDP were included in this study. Finally, 62 patients underwent RVP-SPDP (splenic preservation rate: 83.8%) and the remaining 12 patients underwent robotic-assisted distal pancreatectomy with splenectomy. The reasons for unplanned splenectomy included severe tumor adhesion to the splenic vessels in 6, severe tumor adhesion to the splenic hilum in 5, and severe pancreatitis in 1.

**Fig. 1:**
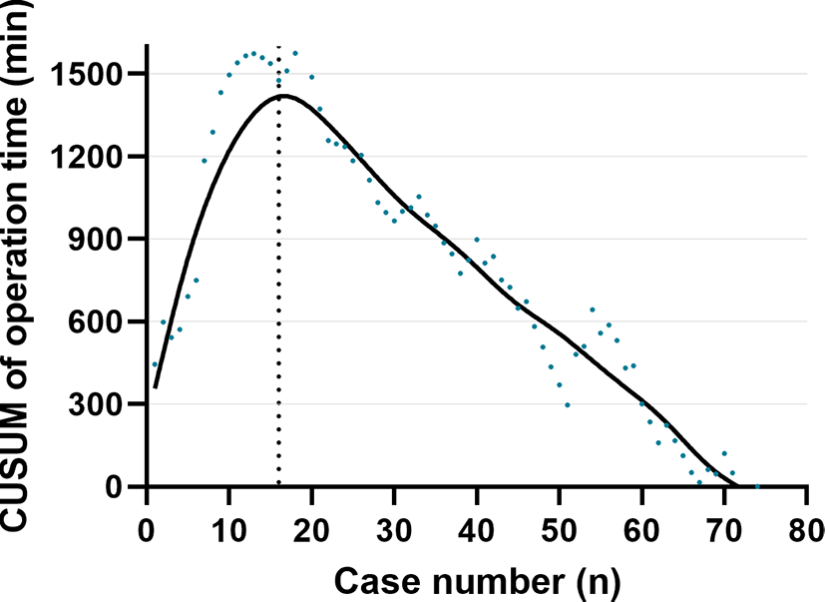
Learning curve of robotic-assisted splenic vessel-preserving spleen-preserving distal pancreatectomy. The inflexion point of the operation time was around case 16

The clinicopathological features of patients were summarized in Table 1. The learning curve of operation time (Fig. 1) was analyzed. The inflexion point was around case 16. Therefore, the patients were divided into the group 1 (cases 1–16) and group 2 (cases 17–74). There was no significant difference in the baseline features of patients, including age, sex, body mass index (BMI), American Society of Anesthesiologists (ASA) classification, tumor size, and tumor pathology.

**Table 1:** Clinicopathological characteristics of patients who were planned to receive robotic-assisted SPDP with splenic vessels preservation

Features	Total (*n* = 74)	Before the inflexion point (*n* = 16)	After the inflexion point (*n* = 58)	*P* value
Age, years, median, (IQR)	46 (35–56)	49 (32–62)	46 (35–56)	0.788^a^
Sex, male, *n*, (%)	25 (33.8%)	6 (37.5%)	19 (32.8%)	0.723^b^
BMI, kg/m^2^, median, (IQR)	24.0 (20.9–25.7)	23.2 (19.7–24.6)	24.6 (21.1–25.9)	0.162^a^
ASA classification I-II, *n*, (%)	62 (83.8%)	14 (87.5%)	48 (82.8%)	1.000^c^
Tumor size, cm, median, (IQR)	2.5 (1.6-4.0)	2.5 (2.0–4.0)	2.4 (1.5-4.0)	0.566^a^
Pathology, *n*, (%)				0.183^c^
Pancreatic neuroendocrine tumor	40 (54.1%)	8 (50.0%)	32 (55.2%)	
Intraductal papillary mucinous neoplasm	5 (6.8%)	1 (6.3%)	4 (6.9%)	
Mucinous cystic adenoma	3 (4.1%)	2 (12.5%)	1 (1.7%)	
Solid pseudopapillary neoplasm	9 (12.2%)	0 (0%)	9 (15.5%)	
Serous cystic adenoma	13 (17.6%)	4 (25.0%)	9 (15.5%)	
Others	4 (5.4%)	1 (6.3%)	3 (5.2%)	

^a^Mann-Whitney *U* test

^b^Chi square test

^c^Fisher exact test

*Abbreviations*: SPDP, spleen-preserving distal pancreatectomy; BMI, body mass index; ASA, American Society of Anesthesiologists

### Short-term and long-term outcomes of patients undergoing robotic-assisted SVP-SPDP

The operative details and short-term outcomes of patients who were planned to receive RSVP-SPDP were summarized in Table 2. The median operation time was 243 (IQR: 195–306) minutes. The CR-POPF rate was 21.6% (grade B POPF: 16/74, 21.6%; no grade C POPF). None of the patients had splenic infarction or abscess postoperatively. The postoperative major complication rate was 4.1%. Three patients were graded as Clavien-Dindo classification grade IIIa due to percutaneous drainage for peritoneal effusion. The median postoperative length-of-stay was 8 (IQR: 7–10) days.

**Table 2:** Operative details and postoperative outcomes of patients who were planned to receive robotic-assisted SPDP with splenic vessels preservation

Features	Total (*n* = 74)	Before the inflexion point (*n* = 16)	After the inflexion point (*n* = 58)	*P* value
Spleen preservation rate	83.8%	87.5%	82.8%	1.000^c^
Operative time, minutes, median, (IQR)	243 (195–306)	318 (256–404)	220 (190–295)	< 0.001^b^
Intraoperative blood loss, mL, median, (IQR)	50 (30–50)	50 (35–175)	50 (30–50)	0.012^b^
Blood transfusion, *n*, (%)	4 (5.4%)	2 (12.5%)	2 (3.4%)	0.202^c^
Division of the pancreas, *n*, (%)				< 0.001^c^
Ultrasonic scalpel	53 (71.6%)	4 (25.0%)	49 (84.5%)	
Stapler	21 (28.4%)	12 (75.0%)	9 (15.5%)	
Postoperative major complication^a^, *n*, (%)	3 (4.1%)	1 (6.3%)	2 (3.4%)	0.524^c^
CR-POPF, *n*, (%)				1.000^c^
Grade B	16 (21.6%)	3 (18.8%)	13 (22.4%)	
Grade C	0 (0%)	0 (0%)	0 (0%)	
Splenic infarction or abscess, *n*, (%)	0 (0%)	0 (0%)	0 (0%)	NA
Postoperative LOS, days, median, (IQR)	8 (7–10)	10 (8–14)	8 (7–10)	0.120^b^

^a^Severity ≥ Clavien-Dindo classification grade III

^b^Mann-Whitney *U* test

^c^Fisher exact test

*Abbreviations*: SPDP, spleen-preserving distal pancreatectomy; CR-POPF, clinically relevant postoperative pancreatic fistula; LOS, length-of-stay; NA, not available

### Improvements in perioperative outcomes of patients undergoing robotic-assisted SVP-SPDP

**Fig. 2:**
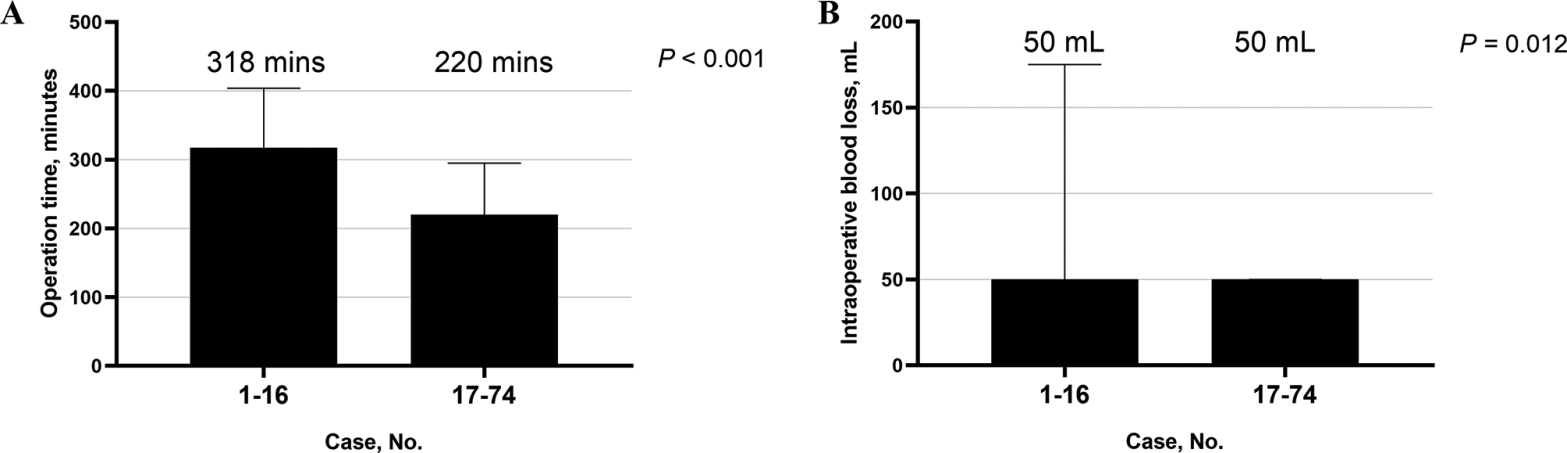
Improvement of operation time (**A**) and intraoperative estimated blood loss (**B**) in robotic-assisted splenic vessel-preserving spleen-preserving distal pancreatectomy

**Fig. 3:**
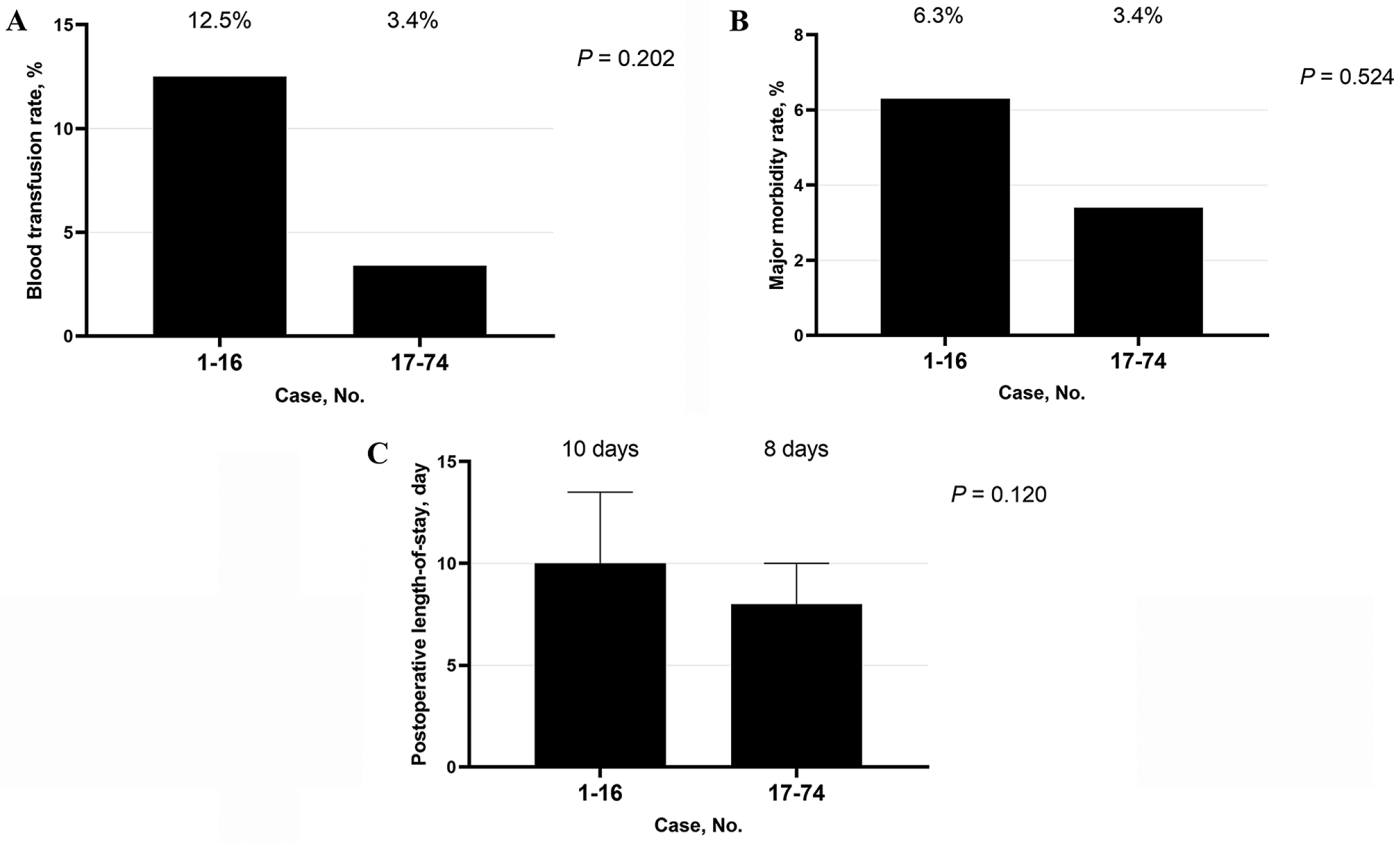
Comparison of blood transfusion rate (**A**), postoperative major complications rate (**B**), and postoperative length-of-stay (**C**) in robotic-assisted splenic vessel-preserving spleen-preserving distal pancreatectomy

It showed that the operation time (median, 318 vs. 220 min; *P* < 0.001; Fig. 2A) and the intraoperative blood loss (median, 50 vs. 50 mL; *P* = 0.012; Fig. 2B) improved significantly after 16 cases. Blood transfusion rate (12.5% vs. 3.4%; *P* = 0.202; Fig. 3A), postoperative major morbidity rate (6.3% vs. 3.4%; *P* = 0.524; Fig. 3B), and postoperative length-of-stay (median, 10 vs. 8 days; *P* = 0.120; Fig. 3C) improved after 16 cases but did not reach statistical difference.

## Discussion

Owing to the increased detection rate of benign or low-grade malignant tumors in the pancreatic body and/or tail, SPDP is favored and performed by more surgeons [17, 18]. In addition, more centers have become proficient in performing MI-SPDP with advances in minimally invasive techniques. An international retrospective study demonstrated that MI-SPDP was associated with less blood loss, less abdominal abscesses, and less splenic infarctions than open SPDP [19]. Besides, robotic-assisted SPDP provides more advantages than laparoscopic SPDP, including improved spleen preservation rate, reduced intraoperative blood loss and blood transfusion [7, 8, 20, 21]. Nevertheless, no statistically significant differences in spleen preservation rates were reported between laparoscopic SPDP and robotic-assisted SPDP after crossing the learning curve (> 16 cases) [22]. However, robotic surgery also has some disadvantages, such as high surgical costs and lack of force feedback. The learning curve of robotic-assisted SVP-SPDP remains unreported to date. Therefore, the current study sought to evaluate the postoperative outcomes and learning curve of robotic-assisted SVP-SPDP by one single surgeon in our institute.

In this study, the operation time, intraoperative blood loss, CR-POPF rate, and postoperative major complication rate was comparable to the previously reported literature [1]. CUSUM analysis showed that significant improvement in operation time and intraoperative blood loss was achieved after 16 cases. The reduction in operation time is associated with shorter docking and undocking times, more skilled operations, and optimized surgical procedures. The median docking time before and after the inflexion point was 23 min and 16 min, respectively. The reduction of robotic docking time is mainly due to the increased proficiency of the assistant in placing the trocar and the operating team in adjusting the position of the robotic system. The reduction in postoperative length of stay was associated with a lower rate of major complications, although the differences did not reach statistical significance. In this study, the CR-POPF rate was 21.6%, all of which were grade B POPF and no grade C POPF. The CR-POPF rate was comparable to the previously reported study [7]. The main cause of pancreatic fistula may be the soft texture of pancreas in patients with benign or low-grade malignant pancreatic tumors [23]. Most CR-POPF only require continuous drainage until removal without additional treatment. Only 4.1% of patients required percutaneous drainage because of abdominal fluid collection. The advantage of SVP-SPDP over SVS-SPDP is the lower incidence of splenic infarction and regional portal hypertension after the procedure [24]. In this study, none of the patients had splenic infarction or regional portal hypertension during the postoperative follow-up. Collectively, this study revealed the safety and feasibility of robotic-assisted SVP-SPDP.

The present study had several limitations. First, this study was a single-center, retrospective study with a relatively small sample size, which may lead to biased results. Second, robotic-assisted SVP-SPDP may not be suitable for large pancreatic tumors close to the splenic hilum or tumors invading the splenic vessels. Most of the cases are low BMI cases, which were considered as easy-to-handle cases. In Europe and the United States, where there are many patients with high BMI, this procedure may be difficult. The long-term outcomes of robotic-assisted SVP-SPDP needs to be further explored by larger clinical trials.

## Conclusion

In summary, this study revealed that robotic-assisted SVP-SPDP was a safe and feasible approach for selected patients after learning curve. The improvement of operation time and intraoperative blood loss was achieved after 16 cases.

## Data Availability

All data generated or analyzed during this study are available from the corresponding author on reasonable request.
